# Haemophagocytic lymphohistiocytosis caused by *GATA2* deficiency: a report on three patients

**DOI:** 10.1186/s12879-024-09356-3

**Published:** 2024-05-10

**Authors:** Lin Wu, Jingshi Wang, Deli Song, Yahong You, Zhao Wang

**Affiliations:** Department of Haematology, Beijing Friendship Hospital, Capital Medical University, No.95 Yongan Road Xicheng District, Beijing, China

**Keywords:** Haemophagocytic lymphohistiocytosis, *GATA2*, EBV, *Nontuberculous mycobacterium*, Myelodysplastic syndrome

## Abstract

**Background:**

Haemophagocytic lymphohistiocytosis (HLH) is a syndrome that occurs in patients with severe systemic hyperinflammation. GATA binding protein 2 (*GATA2*) is a transcription factor and key component in haematopoiesis and stem cell biology.

**Case presentation:**

Three patients with HLH, one with *Mycobacterium avium* infection, one with Epstein–Barr virus (EBV) infection, and one with *Mycobacterium kansasii* infection, were all subsequently found to have a defect in the *GATA2* gene through genetic testing.

**Conclusions:**

*GATA2* deficiency syndrome should be considered in patients with myelodysplastic syndrome, *nontuberculous mycobacterium* infection and HLH. In addition, the *GATA2* gene variant may be a genetic defect that could be the cause of the primary HLH. However, further studies are needed to confirm the role of *GATA2* pathogenic variants in the pathogenesis of HLH.

## Background

Haemophagocytic lymphohistiocytosis (HLH) is a syndrome that occurs in patients with severe systemic hyperinflammation. Depending on whether there is an HLH-related genetic abnormality, HLH can be divided into “primary” and “secondary” categories [1]. Primary HLH is caused by impaired lymphocytotoxic function or pathogenic variants associated with inflammatory activity. At present, more than 100 HLH-related genes have been reported, and evidence supporting the relationship of 17 of these genes with HLH is relatively clear. Familial haemophagocytic lymphohistiocytosis (FHL) is caused by autosomal recessive pathogenic changes in genes that encode proteins required for natural killer (NK) cell and CD8+ T lymphocyte cytotoxicity [2]. *GATA2* deficiency is a disorder with a wide range of clinical manifestations, from lymphedema, deafness, and lung dysfunction to miscarriage and genitourinal abnormalities, but is primarily considered a disorder of the immune system and bone marrow abnormalities [3]. *GATA2* deficiency is caused by various heterozygous variants in the *GATA2* gene encoding the zinc finger transcription factor *GATA2*, which plays a key role in the development and maintenance of haematopoietic stem cells. Importantly, most of these variants arise de novo [4]. Individuals with a mutant allele often lose some cell populations, such as B cells, dendritic cells, NK cells, and monocytes, and are susceptible to *Mycobacterium* infections. In addition, these individuals are prone to myeloid tumours, including myelodysplastic syndrome (MDS), myeloproliferative neoplasm (MPN), and chronic myelomonocytic leukaemia [5].

Here, we describe three patients with HLH, one with *Mycobacterium avium* infection, one with Epstein–Barr virus (EBV) infection, and one with *Mycobacterium kansasii* infection. All of these patients were subsequently found to have *GATA2* pathogenic variants through genetic testing.

## Case presentation

**Fig. 1:**
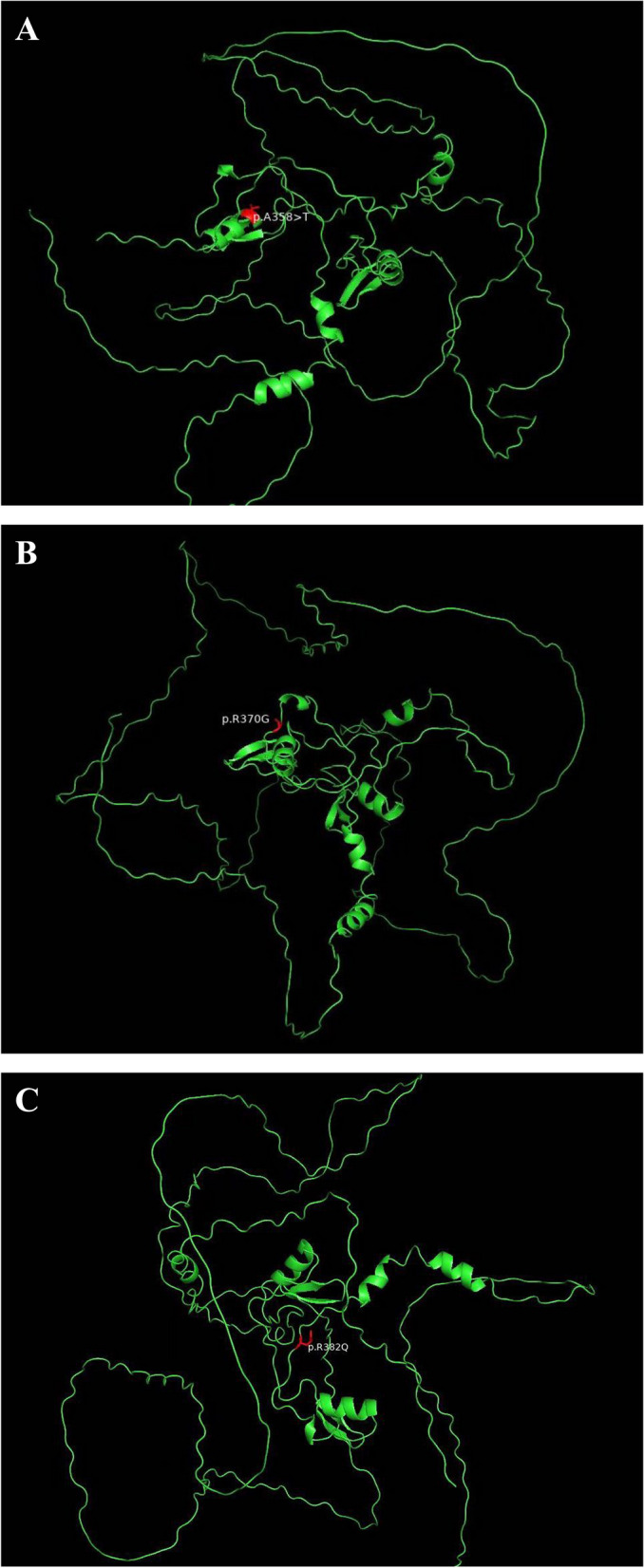
3D-structure of three variants. **A** Patient 1; **B** Patient 2; **C** Patient 3

Patient 1 was male and 28 years old. More than 4 months prior to presentation, the patient had repeated fever without obvious inducement, with a maximum body temperature (Tmax) of 39.5 °C, night sweats, and an increase in body temperature that mainly occurred in the afternoon or evening. Blood parameters were as follows: white blood cell (WBC) count, 1.5 × 10^9^/L; haemoglobin (HGB), 103 g/L; platelet (PLT) count, 63 × 10^9^/L; Monocytes decreased in absolute and relative values; triglycerides (TGs), 2.82 mmol/L; erythrocyte sedimentation rate (ESR), 101 mm/h; and ferritin concentration, 3897 ng/mL. Bone marrow cytology indicated active bone marrow hyperplasia, granulation and red line hyperplasia. There were no typical progenitor cells. Bone marrow flow cytometry revealed a low proportion of mature lymphocytes but no obvious abnormal cells. Chromosome analysis showed 46,XY[20]. Positron emission tomography (PET)/CT examination revealed multiple lymph nodes in the bilateral neck, mediastinum and retroperitoneum with active metabolism and a large spleen with slightly active metabolism. Multiple nodules and patchy high-density shadows in both lungs indicated slightly active metabolism. The patient had lost approximately 5 kg of body weight since onset. NK activity was 11.48% (normal range > 15.11%), and the sCD25 level was 25,825 pg/mL (normal range < 6400 pg/mL). Cytokine testing was performed, and significant increases in MIP-1 alpha, IP-10, IL-6, IL-8, IL-10, IFN-gamma, TNF-alpha, MIP-1beta, and MCP-1 were identified. Therefore, HLH was diagnosed. The patient was positive for EBV-DNA. An anti-tuberculosis test was negative for *tuberculosis* (TB) infection-related T cells. Cervical lymph node pathology revealed suppurative granulomatous inflammation of the lymph nodes, no lymphoma, and acid-fast staining positivity. Polymerase chain reaction (PCR) testing was negative for *tuberculosis* and Epstein-barr virus-encoded small RNA(EBER), but positive for *Mycobacterium avium*. *Mycobacterium avium* was also found in peripheral blood by aetiological next-generation sequencing (NGS).Therefore, *Mycobacterium* infection was diagnosed. Ruxolitinib combined with doxorubicin-etoposide-methylprednisolone (DEP) regimen was given to treat HLH. The *Mycobacterium avium* infection was treated with azithromycin, clofazimine and amikacin. After the 1-year follow-up, the patient’s lymph nodes were smaller than before, and he was negative for EBV-DNA. However, he still had intermittent pulmonary infections, thrombocytopenia, and an enlarged spleen on abdominal computed tomography (CT). No *Mycobacterium avium* was found by NGS (peripheral blood). Whole- Exome sequencing (peripheral blood) revealed a heterozygous missense variant in the *GATA2* gene, and the effect of the variant on protein function was predicted to be harmful (Table 1, Fig. 1). The patient’s daughter (3 years old) had the same pathogenic variant, but no *GATA2* gene variants was found in his parents or older brother.

**Table 1:** Gene loci and variants of the patients

**Patient**	**1**	**2**	**3**
Gene location	chr3.128200691	chr3.128200155	chr3.128200118
Variant	c. 1072 G > A: p.A358T	c.1108A > G: p.R370G	c.1145G > A:p.R382Q
MAF	No such variant was found in 1000g2015aug_all, esp6500siv2_all, ExAC_ALL,gonmAD exome and genome	No such variant was found in 1000g2015aug_all, esp6500siv3_all, ExAC_ALL,gonmAD exome and genome	No such variant was found in 1000g2015aug_all, esp6500siv2_all, ExAC_ALL,gonmAD exome and genome
Polyphen2_HDIV_score	1	0.987	1
Polyphen2_HDIV_pred	D	D	D
Polyphen2_HVAR_score	1	0.78	0.999
Polyphen2_HVAR_pred	D	P	D
CADD_raw	4.433	3.36	4.438
CADD_phred	32	24.5	32
Genotype	het	het	het
Source	DNV	DNV	Unverified
Notes	/	/	His father died of acute leukaemia

*Het* Heterozygote, *DNV* De novo, *D* Probably damage, *P* Possibly damage

Patient 2 was a 22-year-old female. The patient had intermittent fever without obvious inducement 4 months prior to presentation, with a Tmax of 41 °C. Blood parameters were as follows: WBC count, 1.27 × 10^9^/L; HGB, 101 g/L; PLT count, 96 × 10^9^/L; and ferritin, 2630.8 ng/mL. Her liver function test results were as follows: alanine transaminase (ALT), 51.2 U/L; and lactate dehydrogenase (LDH), 610 U/L. The fibrinogen concentration was 3.13 g/L. The ESR was 65 mm/h. Bone marrow cytology indicated obviously active bone marrow hyperplasia and granulation, and the proportion of metamyelocytes was increased. Obviously active erythroid hyperplasia was observed, the proportions of metarubricytes and polychromatic erythroblasts were normal, and piles of nucleated red cells were observed. There were 189 megakaryocytes. In addition, no abnormal cells were found by bone marrow flow cytometry, no myeloid tumor-related genes including *JAK-2* and *BCR/ABL* were found by bone marrow fusion genes. No variants in *ASXL1*, *SF3B1* and *TP53* etc. were found. Chromosome analysis showed 46,XX[20]. Abdominal ultrasound indicated splenomegaly. The EBV-DNA concentration was 9.83 × 10^4^ copies/mL. PET/CT revealed increased nodular metabolism in the right tonsil and multiple nodular abnormalities associated with increased metabolism in the right submaxillary neck, right clavicular region and posterior neck muscle tissue. The spleen is enlarged, and its metabolism is elevated. The metabolism of osteoblasts in the sternum and pelvis was increased slightly in an uneven pattern, suggesting that multiple lesions may be haematologic malignancies. Cervical lymph node pathology revealed histiocytic necrotizing lymphadenitis. She was diagnosed with HLH and EBV infection. Treatment with glucocorticoids and gamma globulin had no obvious effect. Further examination revealed the following: EBV-DNA, 2.6 × 10^3^ copies/mL (plasma) and 2.3 × 10^3^ copies/mL (peripheral blood mononuclear cells [PBMCs]). EBV infection mainly affected B lymphocytes. NK cell activity was decreased, at 7.89%. The sCD25 concentration was 2923 pg/mL. CD107a activation parameters were as follows: NK-△CD107a, 5.16%; and CTL-△CD107a, 6.0%. The expression levels of perforin, granzyme and MUNC13-4 were within the normal ranges. The rituximab-DEP regimen was given to treat HLH. Her condition stabilized, but she was intermittently positive for EBV-DNA after treatment. Three years later, the patient was diagnosed with MDS, and allogeneic haematopoietic stem cell transplantation (allo-HSCT) was proposed. A review of the genetic test results of the patient revealed a heterozygous missense variant in the *GATA2* gene, and the effect of the variant on protein function was predicted to be harmful (Table 1, Fig. 1). No *GATA2* gene variant was found in her parents.

Patient 3 was a 26-year-old male. The patient had fever with fatigue and leukopenia for more than 10 years. Bone marrow cytology suggested that MDS was not excluded. The patient developed chest tightness and fever without obvious inductions 1 month prior to presentation. Routine blood test results were as follows: WBC count, 2.32 × 10^9^/L; HGB, 116 g/L; PLT count, 94 × 10^9^/L; ALT, 195 U/L; total bilirubin (TBil), 22.15 µmol/L; LDH, 704 U/L; and ferritin, > 1500 ng/mL. Bone marrow cytology revealed impaired granulocyte maturation, obviously active erythroid hyperplasia, and polymorphic megakaryocytes. Chromosome analysis showed 47,XY, + 8[2]/46,XY[18]. Genetic testing revealed a *GATA2* missense variant, and the effect of the variant on protein function was predicted to be harmful (Table 1, Fig. 1). *Mycobacterium kansasii* and EBV were detected via NGS. Rifampicin, doxycycline and gentamicin had poor anti-infection effects. The patient’s blood cell counts continued to decline, his ferritin level increased, and his systemic lymph nodes gradually enlarged. Further examination revealed the following: ferritin, 2370 ng/mL; NK cell activity, 14.28%; and sCD25, 16,604 pg/mL. The EBV-DNA levels were as follows: plasma, 3.5 × 10^3^ copies/mL; and PBMCs, not detected. Abdominal ultrasound showed splenomegaly. CT revealed interstitial pneumonia in the right upper lobe and lower lobe of both lungs, with multiple enlarged lymph nodes in the mediastinum, splenomegaly, and multiple slightly engaged lymph nodes in the hepatic portal area. Cervical lymph node pathology and acid-fast staining showed individual cells positive for EBER. The patient had repeated fever, haemocytopenia, increased ferritin, splenomegaly, decreased NK cell activity, and increased sCD25. According to the HLH-2004 diagnostic criteria, HLH, *Mycobacterium kansasii* infection and *GATA2* deficiency syndrome were diagnosed.

## Discussion and conclusions

HLH is a life-threatening disease characterized by a strong immune response that leads to multiorgan dysfunction. HLH usually manifests as a result of familial genetic immunodeficiency diseases or secondary to triggers such as infections, malignancies, or autoimmune diseases [6]. The main factors involved in the development of this disease are an individual’s genetic predisposition to develop HLH, such as a rare associated variant, or an inflammatory process that triggers the immune system to run out of control. GATA binding protein 2 (*GATA 2*) is a transcription factor that is a key component of haematopoietic and stem cell biology. There are approximately 500 published cases containing approximately 180 different familial or de novo germline variants [4, 7].

Due to its rarity, variable clinical presentation, and lack of specific laboratory results, *GATA2* deficiency is often misdiagnosed. *GATA2* deficiency causes heterogeneous abnormalities, as indicated by haematological, immunological, and dermatological findings. *GATA2* deficiency syndromes include cellular immune deficiency disorders, namely, MonoMac (monocytopenia and *Mycobacterium* infection) and Emberger syndrome (myelodysplasia and lymphedema); dendritic cell, monocyte, B and NK lymphocyte (DCML) deficiency; and myeloid malignancies and are mainly associated with familial and primary paediatric MDS and acute myeloid leukemia (AML) [8]. Even within the same family, the age at which symptoms appear can vary greatly, from early childhood to late adulthood. The most common clinical manifestations are present between the second and third decades of life. All three patients were aged 20–30 years (Table 2), which is consistent with the findings of previous reports [4, 5, 9]. Approximately one-third of germline *GATA2* variants are transmitted through autosomal-dominant inheritance (familial), while at least two-thirds of variants are de novo [9, 10]. In this study, two patients had *GATA2* gene variants that occurred de novo, and one patient had a family history of AML, although parental gene verification was not performed (Table 2). Myeloid malignancies are the most common phenotype associated with germline *GATA2* variants, with approximately 80% of reported carriers having a median age of approximately 20 years [11]. All three patients had pancytopenia at disease onset. The first and third patients were not clearly diagnosed with MDS. The second patient was diagnosed with MDS 3 years after the diagnosis of HLH and underwent allogeneic haematopoietic stem cell transplantation due to MDS (Table 2). The first patient was initially diagnosed with *Mycobacterium avium*-related HLH, and the symptoms were relieved after treatment for *Mycobacterium avium* infection and HLH.However, the pancytopenia did not improve, and the diagnosis was made after genetic testing. In conclusion, clinicians should be vigilant for the possibility of MDS in patients with HLH combined with *GATA2* gene variant, as MDS may occur before, at the same time or after the diagnosis of HLH.

**Table 2:** Clinical manifestations of the patients

	Patient 1	Patient 2	Patient 3
Sex	M	F	M
Age (years)	28	22	26
HLH-related indicators			
Fever	Y	Y	Y
Haemocytopenia	Y	Y	Y
Splenomegaly	Y	Y	Y
Ferritin (ng/mL)	3897	2630.8	2370
Haemophagocytic *phenomenon*	Y	N	N
High triglycerides*/*hypofibrinogenemia	N	N	N
sCD25 (normal < 6400) (pg/mL)	25,825	2923	16,604
NK cell activity (normal > 15.11%)	11.48%	7.89%	14.28%
HLH-2004 diagnostic indexes(8/8)	7/8	5/8	6/8
*Nontuberculous mycobacterium* infectio*n*	*Mycobacterium avium*	N	*Mycobacterium kansasii*
EBV infection	Y	Y	Y
NK ΔCD107a (%)	4.97	5.16	/
CTL ΔCD107a (%)	7.6	6.0	/
Monocyte(× 10^9^/L)	0.01	0.01	0.02
Lymphadenopathy	Y	Y	Y
Bone marrow cytology	MDS?	MDS (3 years later)	MDS?

The immunophenotype of *GATA2* deficiency is most often characterized by cellular immunodeficiency followed by recurrent or atypical mycobacterial, viral, or fungal infections. The patients with *GATA2* deficiency syndrome combined with HLH were mostly infected with viruses and *nontuberculous mycobacterium* (NTM) [12–15] (Table 3). In this report, all three patients had EBV infection during the course of their disease; however, unlike EBV-related HLH, EBV infection mainly involved B lymphocytes, and the patients might become negative for EBV-DNA after HLH treatment. Although the pathophysiology of HLH is not fully understood, it is thought that the virus causes an overactive immune response in susceptible patients. Multiple *GATA2* gene variants can lead to diseases associated with haematological and immunological manifestations, such as monocytopenia and B cell and NK cell deficiency. Immune deficiency caused by *GATA2* variant may be the basis of HLH [16]. The resulting *GATA2* deficiency is thought to predispose patients to poor NTM infection control, leading to subsequent persistent immune stimulation in HLH patients. In *GATA2* deficiency syndrome, reduced cytotoxicity of NK cells and specific loss of the CD56bright NK subpopulation have been demonstrated to be associated with impaired differentiation of cytotoxic active NK cells [17]. Moreover, *GATA2* pathogenic variants lead to a decrease in the number and activity of NK cells [17]. In this report, 3 patients with *GATA2* pathogenic variants exhibited a decrease in the activity of NK cells, which is consistent with the findings of previous reports. It is easy to speculate that the low NK cell number and lack of function disrupts the immunomodulatory role of NK cells, increasing the susceptibility to HLH in patients with a defective *GATA2* gene. Thus, HLH may reflect not only impaired infection control but also a genetic predisposition to excessive inflammation. Degranulation damage to cytotoxic T lymphocytes (CTLs) and NK cells was detected byΔCD107a analysis. As previously reported, the quantitative detection of ΔCD107a on the surface of CTLs is highly sensitive and specific for the diagnosis of HLH complicated with granular exocytosis-related genetic disorders [13, 18, 19]. In this report, except for 1 of the 3 patients with *GATA2* pathogenic variant who did not undergo ΔCD107a detection, the ΔCD107a of NK cells and CTLs were decreased in the other 2 patients, as shown in Table 2.

**Table 3:** *GATA2* variant related HLH case reports

**Case**	**Sex**	**Age (years)**	**Gene variant/ locus**	**Inducing agent**	**Symptoms**
**1 **[12]	F	22	c.1009C > T, p.Arg337*	*Cytomegalovirus (CMV)*	Chest pain; cough; fever; malaise; shortness of breath.
**2 **[13]	F	29	c.177C > G, p.Tyr59Ter	*Mycobacterium avium*	Persistent fever; splenomegaly; lymphadenopathy; pancytopenia.
**3 **[15]	F	8	c.1172_1175del, p.E391Gfs ∗ 85	*Varicella zoster virus (VZV)*	Abdominal pain; an erythaematous, vesicular rash; subfebrile body temperature; respiratory insufficiency; bicytopenia.
**4 **[15]	M	7	a c.(16 bp tandem repeat in exon 4), p.T347fs)	*Varicella zoster virus (VZV)*	Fever; rash; cough; oral aphthosis; recurrent furunculosis; hepatosplenomegaly; chickenpox.
**5 **[14]	F	27	c.1061 C > T (T354M)	*CMV*	Persistent fever; pancytopenia

The only possible cure for patients with *GATA2* pathogenic variants combined with HLH is allo-HSCT [12, 20]. Nichols-Vinueza et al. reported 59 patients with *GATA2* variant undergoing HSCT [21]. The overall survival (OS) and event-free survival (EFS) at 4 years were 85.1% and 82.1% respectively [21]. Patient 1 and patient 3 could not undergo allo-HSCT due to economic reasons, and allo-HSCT was proposed for patient 2. All three patients survived to date, but patient 1 had sustained pancytopenia, repeated infection and recurrent HLH, patient 3 had sustained pancytopenia and repeated infection.

In conclusion, clinicians need to consider the possibility of *GATA2* deficiency syndrome in clinical patients with MDS, recurrent *NTM* infection and HLH as the main clinical manifestations, and the *GATA2* pathogenic variants may be the cause of the primary HLH. However, further studies are needed to confirm the role of *GATA2* pathogenic variants in the pathogenesis of HLH.

## Data Availability

The variation data reported in this paper have been deposited in the Genome Variation Map (GVM) in National Genomics Data Center, Beijing Institute of Genomics, Chinese Academy of Sciences and China National Center for Bioinformation, under accession number GVM000731.

## Abbreviations

ALT: Alanine transaminase
CT: Computed tomography
CTL: Cytotoxic T lymphocyte
ESR: Erythrocyte sedimentation rate
FHL: Familial haemophagocytic lymphohistiocytosis
*GATA2*: GATA-binding protein 2
HLH: Haemophagocytic lymphohistiocytosis
HGB: Haemoglobin
HSCT: Haematopoietic stem cell transplantation
LDH: Lactic dehydrogenase
MDS: Myelodysplastic syndrome
MPN: Myeloproliferative neoplasm
NGS: Next-generation sequencing
NK: Natural killer
PCR: Polymerase chain reaction
PET: Positron emission tomography
PLT: Platelet
TB: Tuberculosis
TBil: Total bilirubin
TG: Triglyceride
WBC: White blood cell
